# Genetic ancestry and the colonial legacies of race in genomics: a cross-disciplinary dialogue

**DOI:** 10.3389/fgene.2024.1523406

**Published:** 2025-01-23

**Authors:** Ernesto Schwartz-Marin, Tayyaba Jiwani, Sarah Abel, Yulia Egorova, Amade M’charek, Diogo Meyer, Andrés Moreno Estrada, Katharina Schramm, Peter Wade, Michel Naslavsky

**Affiliations:** ^1^ Department of Sociology, Philosophy, and Anthropology, University of Exeter, Exeter, United Kingdom; ^2^ Institute for Philosophical Research, National Autonomous University of Mexico, Mexico City, Mexico; ^3^ Department of Anthropology, University of Durham, Durham, United Kingdom; ^4^ Department of Anthropology, University of Amsterdam, Amsterdam, Netherlands; ^5^ Department of Genetics and Evolutionary Biology, Institute of Biosciences, University of São Paulo, São Paulo, Brazil; ^6^ Advanced Genomics Unit, Center for Research and Advanced Studies (Cinvestav), Mexico City, Mexico; ^7^ Faculty of Humanities and Social Sciences, University of Bayreuth, Bayreuth, Germany; ^8^ Department of Social Anthropology, University of Manchester, Manchester, United Kingdom; ^9^ Department of Genetics and Evolutionary Biology, Biosciences Institute of the University of São Paulo, São Paulo, Brazil; ^10^ Hospital Israelita Albert Einstein, São Paulo, Brazil

**Keywords:** decolonial, race, sovereignty, hetero-identification, genetic ancestry, population sampling, racialization

## Abstract

As genomics initiatives have spread around the world–often in the name of genetic diversity and inclusion–they have not only invoked promises of a medical revolution, but also revived categories of human difference that resemble erstwhile racial classifications. This is despite the fact that geneticists broadly dismissed racial categories as obsolete and unfounded after the Human Genome Project was completed in 2003. In fact, contemporary genomics initiatives have often ended up reinforcing ethnocentric and nativist conceptions of difference, drawing intense criticism from activists and critical social scientists. This roundtable brings leading population geneticists grappling with the question of genetic identity and ancestry, especially in the global South, together with some of the most prominent scholars of race in genomics. The result is an engaging and insightful dialogue on questions that have vexed the field for decades. How do we—indeed “can” we reconcile the boundaries of biological and social difference? How do notions of “genetic ancestry” and “biogeographical ancestry differ from erstwhile racial and ethnic categories? Can racial categories ever be shorn of their colonial and oppressive legacies? Here we scrutinise the methodological and epistemological frameworks in contemporary genomics that work to define populations and shape our understanding of biology, society, health, and disease. We seek to clarify perspectives across the disciplinary divide, and to advance constructive and grounded critiques that contend with the question of justice in genomics.

## Biogeographical ancestry and race

**Ernesto Schwartz-Marin:** Let’s begin this conversation with a very simple question. What is the difference between race and biogeographical ancestry?

**Peter Wade:** I can start. My sense is that most geneticists would say there is no relationship—race is one thing and geographical ancestry is completely different. Mainly because the genetic markers typically used to trace biogeographical ancestry happen to be present in a given population for evolutionary reasons and do not necessarily have any relationship to phenotype, let alone skin colour, hair form, and facial features—you know, the usual markers of race as we understand it in everyday language. So, in that sense, they’re taking these particular genetic variants, which are more common statistically in one population than another, and using them to trace ancestry. But I do know that a minority of geneticists—David Reich is the most famous, I suppose—say that the distribution of these genetic variants is related to the attributes we’ve traditionally called “race”. Perhaps some of our geneticist colleagues here can tell us what the latest thinking is along these lines.

**Sarah Abel:** Speaking from the social sciences perspective, we have an increasingly clear idea of how to define race. I’ll put out a tentative definition: race is a social and historical construct (not a biological one) which was used, and continues to be used, as a tool of colonization, a means of categorising and ordering bodies in order to create differentiated structures of oppression.

But obviously things are not as easy as that. Race in the colonies was also *made,* for example, through reproduction. So when you’re looking at genetics, you’re also seeing a history of sex, and there’s indeed an entanglement there between the cultural and the biological.

I’m also very interested in how people who engage with genetic technologies read race *into* them. For instance, we can see how commercial DNA ancestry tests have evolved to try and cater to perceived consumer demands and notions about the relationship between genetics, race, and identity. Around the 2000s, test results would be displayed as large biogeographical or continental categories, but these did not always correspond to what people expected with regards to their own sense of race. Over the course of 15–20 years, DNA ancestry testing companies realised that what was most interesting to customers was a test whose results would look like an identity they recognised. So they evolved their tests to more strongly align with racial, and to a certain extent ethnic, identities particularly as they are expressed in the United States, where a large proportion of the companies and their customers are based.

**Diogo Meyer:** There are two points I want to share, especially from the context of Brazil. The first is dealing with the observation that conventional racial categories and genetically defined groups (or biogeographical ancestry) *are* correlated to some extent. There are technical reasons for this, including the history of human movements or isolation by distance. But I find it a recurrent difficulty to explain to people that racial categories and genetically defined groups are different things in the way they are constructed, perceived, and used, and yet may be correlated in an almost trivial quantitative sense.

The second point is that even when you’re talking to geneticists who accept the limitations of racial categories in describing genetic diversity, they sort of rely on them. In Brazil they are very common in medical culture. In the census, Brazilians are all classified by race—Black, White, Pardo (to convey an idea of a colour “in between” black and white), Yellow (which has stuck in our literature and refers to an Asian origin), and Indigenous.

People will often say that in the absence of accurate biogeographical ancestry information, which we might want to use in biomedical research, we have to resort to using race as a proxy. This idea of “race as proxy”, that it is better than nothing, is very prevalent in the medical community. My general response is that it needs to come with a huge warning or disclaimer. The proxy can sometimes by useful (e.g., in prioritising groups to recruit for transplantation, as we have shown (Nunes et al., 2020)), but it comes with a huge cost in terms of naturalising the concept of race.

**Michel Naslavsky:** I’d like to complement what Diogo said about the Brazilian context. We have plotted ancestry against those census classifications in a sample from São Paulo, and there *is* a correlation. It does not mean that the concepts are dependent on each other, but there is a correlation between them. And this, I believe, is a consequence of the demographic history of Brazil, meaning that the practices that Sarah defined very well are real.

Also, Brazil has had university admissions quotas based on self-declared race/ethnicity definitions for the past 20 years. And they do, in fact, proxy many other socioeconomic positions and disadvantages as a result of Brazil’s history. This means that biogeographical ancestry is indeed conceptually uncoupled, but there is a correlation with race and this has wider implications.

**Amade M**’**charek**: I think one of the mistakes we should try to avoid is thinking that biogeographic ancestry is a given, that it is something that’s “out there”, while “race” is constructed. I want to question this. I remember that in the early days of the Human Genome Diversity Project, the late Allan Wilson argued that all definitions of population are arbitrary and that we sample populations based on a grid approach: take a sample every 100 miles. This was you could side-step any social clustering or “natural” barriers between people, be they rivers, mountains, etc. And of course we *did not* take that route.

So it would be important to pay close attention to the way databases have been set up, the way we have clustered people based on existing social, economic, historical, natural borders, and its effects. Because this becomes self-reinforcing, right? We’re first producing a particular kind of diversity and then we’re returning to it to prove it exists. I do not want to oversimplify, but this is a key issue to examine.

Second, I echo what Sarah has said about the historical weight of the concept of race. I’ve been working on race for ages and recently, I sort of saw the light. I’ve been analyzing a scientific paper on facial morphology, and I wanted to figure out why it seems we are talking about different things—for example, people saying they are not working on race but social scientists showing that in practice they are … I really wondered what was going on.

Then, by looking into the multiple ways that differences are *made*—from sampling to the ordering of data and then its analysis—I figured out that really, we have three different phenomena that we call “race” as if they are the same. The first is about clustering people and their bodies, by color, religion, culture, what have you; we’ve done everything actually. So there is no obvious definition of what race *really* is. The second is the use of race as a methodological tool: mobilizing assumed differences to order and categorise data that you work with. And the third is a more theoretical approach where you rely on admixture and of course, evolutionary theory and mutation rate, etc. to analyze your results.

For me, this was really an eye opener. And it might be helpful in trying to figure out the matches and mismatches between biogeographic ancestry and race. This keeps on the table, of course, the political weight of race. Race is a political thing, we must remember—we cannot do away with that history.

**Katharina Schramm**: My thoughts are in a similar direction. Your question suggests that biogeographic ancestry describes what was formerly called race. That there may be an “evolution” in the biological sciences, from race to a more “accurate” form of classification, which matches human variation in a better way. And geneticists would probably say yes it does, because it is more differentiated, and so on.

But as Amade has described, there are these haunting reverberations of classificatory practices, of historical hierarchies, of the violence that surrounds race—not only in the terminology we use, but also in the practices around race and race-making (e.g., Graves, 2023). And this is where STS or critical social science has engaged with genomics, tracing out these ghosts, cautioning not to consider any classificatory practice as “neutral” or simply “out there” in nature.

The reference to the sampling grid is really interesting. I would be very interested in discussing how sampling is done today. From what I saw in South Africa, population genetics sampling relies on the idea of the “third generation” [i.e., stable generational links with established racial/ethnic identities]. It relies on ethnic markers, on social categories that build on legacies of race. And what does not fit, does not enter the sample. So it is somewhat a self-fulfilling prophecy.

**Peter Wade:** Just as a parenthesis, I think it is important to remember *why* geneticists are looking at things like biogeographical ancestry; what it is they’re trying to achieve. And in many cases, it is to do with medical genetics, i.e., controlling for population stratification and so forth. So in some ways, we have to acknowledge that geneticists are trying to do something different from us anthropologists or social scientists. For many geneticists, biogeographical ancestry is a kind of handy tool to achieve some other objective. I’m not quite sure how to resolve this, but it is important to acknowledge.

But equally, we’ve found in our research that even though geneticists, including people like Sérgio Pena in Brazil, spend much time denying the biological validity of the concept of race, they often use simple and crude ideas of “African”, “European”, and “Indigenous” biogeographical ancestry. The way that further gets represented in graphs, tables, etc. is in very simple (and potentially problematic) ways. Pena, for example, would produce these genetic ancestry diagrams with “Africa”, “Europe” and “America”—they’d just have those words there. And he’d place his samples relative to their genetic distance, from these supposedly “pure” points of origin. These kinds of representations feed extremely easily into people’s everyday concepts of “la raza indígena”, “la raza africana”, “la raza blanca”, and so on. So, a lot of this is also about the way these things get represented in the public sphere.

I also agree with Amade and Katharina, that the underlying problem is the concept of “population”, and the way that it is defined in social terms but investigated in biological terms. As soon as you do that, you cannot help but create a biological genetic profile for a socially defined group. It is just inevitable.

**Ernesto Schwartz-Marin**: Thanks a lot for that, Pete. I like how you framed that–populations defined in social terms but investigated in biological terms.

**Andrés Moreno**: I want to add to Pete’s comment and his earlier example of some geneticists saying that genetic results in fact confirm racial classifications (David Reich, for instance). I think it is clear that although biology and genetics have been demonstrating for decades that races do not exist from the biological point of view, there is an emerging narrative or perception that, “Wow, now geneticists are finding that indeed, their results kind of reinforce racial groupings.’

I think this is a problem of interpretation—the fact that we see differences between human populations does not demonstrate that *races* exist. The vast majority of evidence still shows that we share more than 99.9% of our DNA, so there’s no biological basis for race, as currently defined. But that does *not* mean that we cannot direct our focus to the very interesting points of difference between human populations. And while looking at those differences, people sometimes take a simplistic approach to categorizing them, because it is convenient to say that one identified a “European population” as opposed to African, and so on.

However, it is important to note that those labels, even if they are as simple as geographic, are discrete endpoints on a larger gradient, which is what our studies have been discovering. There is no “pure” population only existing in Africa, Europe, Asia, or the Americas. We have been learning that there’s a gradient of genetic variation, with no defined limits that demarcate where population A ends and population B begins.

We should try to reflect this gradient in our nomenclature, in the way we report science, and so on. In fact, this variation is what biology is like—what changes is the way we scientists try to understand it.

And that goes back to another point you made, Peter, that it is inevitable to attach some categorization or label if you want to refer to a group, whether it is geographic or ancestry-based, and that has its problematic entanglements. So, we’re still figuring out how to refer to things in accurate and representative ways, while ensuring that we enter public discourse in more equitable ways.

## Race, racial identity, and racism

**Ernesto Schwartz-Marin**: I want to be the devil’s advocate here. I think that biogeographical ancestry does not do a lot to move beyond race; actually, it reinforces a lot of ideas of continental difference. And on top of that, it tries to sanitise and distance itself from the political implications of thinking in terms of continental variation.

What I’m saying is that “races” did not exist before the colonial encounter. Before colonialism, in Latin America we had the Aztecs, the Chibchas. and so on. Even though physical differences among them were recognized, these very racial logics of organising society are colonial through and through. And biogeographical ancestry does not do a lot to move away from that.

I like Pete’s comment that we are doing different things. Geneticists are trying to think about biogeographical ancestry, but as Amade was saying, the way we “do” race in social science is very different. However, I have the sense that geneticists do not necessarily *need* biogeographical ancestry to deal with lots of medical issues they work on. I spent 2 years in a Mexican lab where they studied fatty liver and Indigenous people, but they could just as well do away with the Indigenous category and talk about all the different ethnic groups that share a haplotype.

In fact, in our ethnography in Colombia, lots of geneticists were tired of engaging with these racial categories. They wanted to do something more interesting. I really liked your new paper, Andrés, in which you talk about different Indigenous groups, but still you feel that biogeographical ancestry is slipping in there somehow or another, right? Because you need to connect with certain audiences, journals, etc. Another problem is: What other categories do we have to deal with biological diversity?

**Yulia Egorova:** Building on your comment, I’d like to point out that different new languages and concepts that have emerged in the “post-race” space also do not do much other than contribute again to essentialized thinking about human diversity, connecting cultures to some perceived biological reality “out there”. For instance, we can look at debates about the origin of osteology (bone record) collections, particularly in cases where we’re really not sure and lack historical records showing us exactly where they came from. Once we start relying on knowledge from genetics or forensics, we keep falling back on existing categories, whether we’re talking about races, populations, or regional diversity. We end up with a very essentializing construction.

And this essentializing language is found not only among geneticists, forensic anthropologists and other biological scientists, but also activists. We can look at processes of repatriation of osteology collections—on the one hand, of course it is absolutely fair, it is about addressing injustices, but on the other, the vocabulary of activists also relies on the construction of racialized identities and ends up reinforcing them.

So part of me feels that maybe we need a completely different conceptual apparatus. Maybe sometimes emphasizing uncertainty of origins and de-emphasizing any possible connections between cultural categories and biological categories is important. How we may develop such a conceptual apparatus is a separate question. But it is clear that we’re struggling with the language we have at the moment, where we keep falling into the trap of reinforcing a perceived connection between cultural populations and the biological realities allegedly standing behind them.

**Sarah Abel:** Tying into Diogo’s point that in Brazil the racial categories used in medical genetics are colour categories, based on census categories, there’s something specific to be studied there. How did those categories come about? Why are census categories being used? It is important to understand the history of these census categories, which are in many ways arbitrary as well.

In fact, in Brazil, there has been much back-and-forth about the relationship between colour and genomic ancestry. Geneticists like Sérgio Pena and Maria Caítra Bortolini were at the forefront of these discussions back in the 2000s in the context of affirmative action debates. Pena, in particular, argued that since everyone’s mixed in Brazil, you can be any colour, any phenotype, and one could not guess your genomic ancestry (Pena et al., 2000; Pena and Bortolini, 2004; Pena, 2009). These geneticists—like many other Brazilians—were very invested in the idea of mestiçagem (a homogenously mixed or hybrid population) as a democratizing force in Brazil, and this is a point that needs to be thought about critically.

But also, I think geneticists need to think what are their intentions for race as a political category, and how they are engaging with that in their research? We’ve talked almost exclusively about race so far, but it is important also to think about racism—i.e. what is the relationship between race as a category and racism.

For medical genomics, I think racism ought to be more important than race or biogeographical ancestry. There are some interesting studies, for example, on how structural racism *creates* medical phenomena (Gravlee, 2009; Graves and Goodman, 2021; Krieger, 2021). It produces pathologies and interacts with the onset of diseases. This does not necessarily have to do with ancestry--it is not coming from inside the body, but from outside: from people’s experiences of racism as violence and stress; from racialised segregation which exposes bodies to toxic environments; from a structural lack of access to healthcare and social support networks.

And going back to Brazil, colour may be more relevant than ancestry when we’re thinking about racism because that’s usually the way that people are racialized and experience racism. So, it is important to be precise, and think about what is really the phenomenon (race, racism, etc.) that is relevant for the sort of research that we’re doing.

**Peter Wade:** One of the reasons, I think, why we social scientists get so worked up when geneticists talk about biogeographical ancestry, is that we think this will end up reproducing familiar notions of race in a different language. We assume that doing so will exacerbate racism.

And that’s an empirical question. What happens to concepts of biogeographical ancestry when they get out into the world? What work are they doing there? Do they increase people’s tendency to be racist? Well, I’m not sure. I do not think it is quite as clear cut as we might assume.

On the other hand, if we look at what happened in Brazil with Sérgio Pena—he spent a lot of his time challenging the idea that race was a biological reality, but his data were being used in the debates about affirmative action to challenge the idea that you should be directing social policy towards a so-called Black category. Because he said, “Well, everybody’s mixed in Brazil. Genetically, there’s no such thing as a Black category. Social policy should follow biological reality” (Birchal and Pena, 2011). That was his, you know, illogical view. But that was actually a racist consequence of the use of genetic data, ironically one that came from saying that race does not exist as a biological reality.

**Sarah Abel:** I completely agree with Pete; that’s a really important point. I think there is a sense of “Would not it be convenient if biogeographical ancestry was just a non-problematic category, a shorthand and you did not have to think about it anymore?”

## National genomes and nativism

**Tayyaba Jiwani:** I wanted to touch back on the point that if we analyse populations by biogeographical groupings, we do see some differences that are consistent, which indicates that those ancestral groupings might be “real” biologically. And I want to push back on this, because the fact is that our final results are so dependent on our initial starting points, right? As others have mentioned, the sampling practices that defined the first founder populations continue to determine our subsequent analyses so that we continue to reinforce those definitions.

For example, rather than dividing populations by continental groupings (which of course comes from a certain colonial epistemology), if we had divided populations by class or say caste, we would have arrived at a completely different genetic grouping of human populations. And if we continued to use that overarching framework to interrogate contemporary populations, we would have seen consistent genetic differences between different class and caste groups, to infer some kind of biological reality.

And certainly this becomes quite clear with the emergence of purported “national genomes”. Nations carved out of populations that were mixed until very recently, for example, in South Asia, are asserting their unique identity by establishing unique patterns of genetic variation. And of course if you take two populations as given, you may be able to find patterns of genetic variation more common in one than the other. But that does not legitimise any inherent biological distinction between the two.

**Ernesto Schwartz-Marin:** Pushing on this provocation further, do you think genomics is nativist by design? By nativist, I mean the narrative, legal, scientific, and political frameworks that reinforce the idea that people are truly “naturally located” in some places, belong inherently to some particular land.

We’ve talked about the third generation and such ways of defining identity and belonging by birth—I, for instance, cannot be part of the “Mexican genome” because one of my ancestors was a White man, a Jewish refugee, while others were Indigenous people from different parts of Mexico. Because of this White man, I’m not “pure” enough to be part of the Mexican Mestizo sample in the third generation, which is strange, because I should be mestizo in any way or form. So, I believe that genomics is also quite a nativist project because it starts with the sampling of “native” populations and then reproduces them. What do people think about this?

**Peter Wade:** I’m not sure I agree because, as you know, what we found above all in Brazil, Mexico, and Colombia was that their national populations were represented as “mixed” populations. And you’d be a very typical example of that as someone of Indigenous and European ancestry—a typical mestizo Mexican. So yes, it is nativist in the sense that you construct this pure idea of the original Indigenous population against which you measure “mestizaje” (or mixture). But I do not think genomics is inherently nativist; it can be used in a nativist way.

Some genetics projects done in the United Kingdom did create a kind of nativist impression of Britain as a genetically heterogeneous place, but in a way that excluded recent migrants—all the people who came to Britain after around 1945 were *not* part of that heterogeneity. They were somehow outside it. So although heterogeneity was admitted, it was a nativist version of heterogeneity. A certain kind of mixture was constituted Britishness.

**Andrés Moreno:** You’ve raised an interesting example, Ernesto. I think it depends on the methodology or purpose of the study. If the sampling design is to capture the ancestral lineages of a given place or population, it might make sense to narrow down the participation of individuals to those that are actually representative of such lineages. And this is typically done by ascertaining two or three generations back in time.

But I think in most modern genomic studies, this should not be an exclusion criterion anymore because now we have the tools to really dissect these mixtures bioinformatically in anybody’s genome. So now it does not matter how mixed you are, or the proportions of the different ancestors, because you can focus on a given part of your genome that is of Jewish ancestry, Indigenous ancestry, or European or African, etc. And that allows us to include as many individuals as possible in the study. So it depends on the study design and purpose.

If it is a national reference study, such as the Mexico Biobank that we’re doing, it includes everybody. It is a census-based design that of course includes rural areas correlated with high proportion of Indigenous people, and urban areas where mixture will be much higher. And that is not a limitation for us to still recover a lot of ancestral civilizational history, and also the profile of the modern population which involves more recent mixture waves, including African, Asian and European populations. Thanks to the technology that we have nowadays, and the methods to do local ancestry deconvolution, there should not be such exclusion criteria anymore.

**Amade M’charek:** I want to say something about biogeographical ancestry and this idea that it is a “given”, something that you can go after through genome wide association studies (GWAS) etc., to figure out how we are all related. In fact, we must remember that this concept is also a construct and it cannot be seen, you know, aloof of all sorts of social, economic, historical differences that have been made. It is important to understand and question the ways in which biogeographical ancestry surfaces in research and published literature. And, indeed, to question if and how it becomes racialized.

Given the persistence of race, I think it is crucial to ask: what is the use of race? What work does it do for us academics or scientists, and what kind of work do we find problematic? So yes, it can function as a proxy, but it is often, really, made to do work for fuzzy categories. The kidney failure test is a famous example, where differences in muscle mass have been translated into those of colour. For people with darker skin colour, a correction is introduced in test results (GFR) before analysing them. What we’re correcting for is differences in muscle mass, but because the clinical eye is not trained to differentiate between bodies in muscle mass, race (in the form of skin colour, ethnicity, national background, etc.) becomes presumably an easier tool to use in practice.

In the Netherlands, for example, we have very slim, dark colored people from sub-Saharan Africa. I read the research in sub-Saharan Africa and this correction is not used there at all. So, it is prevalent in the United States and Western Europe, but not used elsewhere, even in Latin America. And in the Netherlands, until recently the category for race that has been used in clinical practice is “negeroid”, an archaic racist category. So I think we need to deeply think about the politics of race and racialsied categories, and the work they are doing. Whose work are they making lighter? At the same time, it is difficult because they’re also serving as tools to make sense of more difficult categories.

So I think we need to address two things: What exactly is biogeographical ancestry and how it is being used? We need to push for an understanding of it as a socially constructed category, even if it does not look like it. That does not necessarily mean that you should not use it, but you must ask what is the question exactly that is being studied? And the other is perhaps to really problematize race, and at the same time show that it has been mobilized to do the work of communicating difficult messages, while keeping itself alive.

**Yulia Egorova:** Just a quick interjection on your example, Ernesto, with your Jewish ancestry constructed as White—as you probably know, there’s interesting work in Jewish Studies that has challenged the idea that Jewish communities could be subsumed under the umbrella of the White majority. So having geneticists, with the authority they have in the public imagination, say that a Jewish person means White, that is certainly a throwback to discourses that take us politically in a wrong direction.

**Ernesto Schwartz-Marin:** I think you’re right. In Mexico, in those days, there was a constitutional article under which White people were wanted to improve the racial stock of Mexico. So, they accepted my Jewish grandfather and in this specific racial topography he became White—of course he was an Ashkenazi Jew, and they also claim whiteness.

## Problematizing ancestry mapping

**Ernesto Schwartz-Marin:** This brings me back to the question Sarah also raised. Racism and colour, genetics and ancestry, and all this space for contestation, has to do with the ways we “make” race. So maybe one positive intervention in this dialogue is to suggest that every time we use biogeographical ancestry, race or other types of population-making technologies or ideas, we problematize them rather than backgrounding them? This, I think, is something that Diogo mentioned in his first comment.

**Sarah Abel:** I’m remembering what Diogo said at the beginning, that one of their grant proposals went through several rounds of review but no one at any point commented on what would be the ethical procedures around race, and so on.

This highlights an additional problem with how you might “do” race or ancestry in genomics, which is that it is not recognized as something you should spend time or resources questioning or thinking about critically.

There’s often no funding for a social scientist to be on board in these projects, or for any training that would allow geneticists to get more of a grasp on the problems at hand—what they need to be aware of when dealing with such population categories, etc. It would be interesting to hear from the geneticists on the panel on this matter. What are the logistical, structural issues that prevent more critical engagements with these issues?

**Diogo Meyer:** Thanks for these questions. I want to share something about affirmative action in Brazil. I’m not sure whether you are aware, but the recent implementation of affirmative action (and my experience is more from the University of São Paulo) is very explicitly based on a phenotypic definition. As a biologist, you tend to think of phenotype as something highly multi-dimensional, involving where you live, what you do, and so forth, whereas here it is being defined as a physical phenotype (skin colour, head shape, size of lips) and nothing else.

The University of São Paulo is establishing what we refer to as hetero-identification committees, which are responsible for visually validating a person’s status as belonging to a group entitled to an affirmative action policy. The idea is to look at a candidate seeking admission to the university through the quota system, and ask if they’d be perceived as Black in other dimensions of society, including those where they’d be at a disadvantage, for example, in facing police violence which is a major issue in Brazil.

So when I give talks about genetics, people generally feel comfortable that the issue of genetic ancestry is irrelevant to our work in establishing affirmative action, and that they’re dealing with separate issues. They are correlated, as Michel and I discussed earlier, but the correlation is explainable through shared history and does not address what affirmative action is intervening against, which is the racialization of physical appearance. However, I do think it is an interesting issue to think about phenotype being multi-dimensional and not restricted to physical appearance, and I have not really been informed of how this can be dealt with in the context of affirmative action policies.

The second issue is about the bridge between genetics and the social sciences—I’ve been reaching out to people outside my technical circle only recently. I do not think there are explicit barriers, but there are different cultures, different vocabularies, and a lack of incentive from funding agencies.

With the pressure (at least in Brazil) to publish and have your genomics papers out in top-notch journals, the work involved on this “different” front, and the establishment of interactions with a different community, would be seen as something holding you back, taking up precious time and not adding to the “research”. I do not think a book chapter dealing with the issues that we’re discussing around race, which would be time consuming, would be valued in the same way as a technical paper, and therefore funding explains much of this pattern. Interestingly, by requiring such interactions, funding could have a positive effect, in the opposite direction.

**Andrés Moreno:** I was recently part of the United States National Academy of Sciences Engineering and Medicine (NASEM) Committee on the Use of Race, Ethnicity, Ancestry and Population Descriptors in Genomics Research. It was commissioned to discuss how to better use population descriptors in genomics research. It engaged experts from genetics, epidemiology, health disparities research, and others and compiled their insights into a substantial report (National Academies of Sciences Engineering and Medicine, 2023).

This report issued a series of recommendations on using population descriptors precisely because they have been historically used in medical settings, where race has been a handy tool to classify patients as Black, White and so on. It is still used in practice in the United States through the so-called OMB categories, basically categorizing people in ways that have nothing to do with their actual biological background that we’re studying. So, it sets out to reconcile these two approaches.

Similarly, we need refine the use of the term “ancestry”, because we just assume that people would understand its meanings and connotations. We’ve been trying to find the best word to explain that ancestry is just the output of an algorithm which simplifies a model of grouping people. It is not *really* your “ancestors”, but a description of how your genetic profile is being grouped or classified given a certain reference panel. This long explanation is difficult to summarize in a single word, which typically is “ancestry”.

In general, the committee’s recommendation was to move away from the term “ancestry”, in favour of “genetic similarity” or “genetic similarity in the context of a given reference panel”, because that’s how estimation works behind the scenes. You compare a given individual to your reference panel to assign their ancestry proportions, and the sensitivity of your proportions will depend on what you have in your reference panel. This exactly how 23andMe and all these direct-to-consumer companies work.

So, if we say that your ancestry has been estimated by this particular analysis, given this particular panel, that’s more accurate than saying your ancestry is X, Y, or Z. Because ancestry is a reality that you have in your DNA but it includes a broad array of ancestors that our assays are not necessarily capturing. We would need to have a huge reference panel to really capture everybody that has contributed to your ancestry. In sum, ancestry is a reality, but what we report in genetic studies is more a snapshot of analysis that is limited by the reference panel used.

So our committee recommended that in anthropologically driven or population genetics studies, it is better to use the term “genetic similarity”, and maybe there’s room or valid grounds to use racial categories for other types of research, for example, that on health disparities.

**Michel Naslavsky:** I agree with Andrés. I’m following the direct-to-consumer market in Brazil and it is interesting that from time to time, these companies recalculate or update their databases, leading to changes in people’s “ancestry” and they get so upset. So moving to terms like genetic similarity, or the idea that ancestry results are a snapshot relative to a certain reference panel, is a much more feasible way to understand this.

But I also wanted to mention something that came up both from direct-to-consumer company results and from Prof Sérgio Pena. It is something that reinforces the idea of a link between concepts of race and biogeographical ancestry—the comparison of ancestry proportions between mitochondrial or Y-chromosome DNA versus autosomal DNA. In Brazil, there is a large disparity between the proportions, and that’s probably due to known historic patterns of reproduction. For autosomal chromosomes, the ancestry proportions come up to 70% European, 20% African, and 10% Native American on average, while the mitochondrial ancestry is roughly one-third across the board (i.e., a third European, a third African, and a third Native American), and in Y-chromosomes it is 80% European ancestry. This is evidence of disproportionate reproductive patterns which reinforces the known demographic history of Brazil.

So there is a kind of pendular movement between using these categories as strongly or loosely linked, depending on the narrative to be built. And I think that is also a potential place for misleading ideas. This is in the case of Brazil. I’m pretty sure it is not so different from other places in the Americas.

We’ve produced a YouTube playlist “Ancestry ABC” to explain concepts regarding ancestry to the public in Portuguese. It reflects some of the concerns about the over-expectations from genetic testing sold to consumers without good supporting material for them to understand what they’re buying.

**Ernesto Schwartz-Marin:** I wanted to say something around the issue of accuracy. I’m not so sure, Andrés, if being more accurate and emphasizing the uncertainties of our calculations addresses the issue that Amade and others have raised about how race is actually inbuilt in the databases and reference populations themselves. That’s a point that cuts deeper than just being accurate and taking a relativistic approach. I’m also wondering if the committee included any STS scholars doing race and genomics, or was it only scientists like population geneticists and public health officials?

**Andrés Moreno:** Yeah, mostly scientists. I was the only non-US member, though NASEM will of course be biased towards the United States as it is a national body.

**Sarah Abel:** I love the definition that Andrés gave of ancestry as “the output of an algorithm which simplifies a model of grouping people”. I’ve been reading up on AI and algorithmic bias recently, and it is very interesting that a big part of how genetics is done, how estimates are produced, has to do with algorithms and automated technologies. We’ve mentioned how sampling practices create race, but race is also materialized in artifacts like SNP chips, and most of the big commercial players are using SNP chips that are biased towards European populations (Zanetti and Weale, 2018; Abel, 2021). It is not well understood what the implications are for the ancestry results they produce.

I also want to pick up on what you mentioned, Michel, about people getting upset when they see their ancestry results change. I think that really says something about the expectations that people have of genetics getting to some deep truth, an essential truth that should not change, which is not the way actual genetics is done. It is probabilistic, it is to do with statistics and it is contingent on the sample arrays and technologies we have. It would be very interesting for the public to have more of a sense of the contingency of ancestry and the processes of making/remaking race through that.

## Genomics and justice

**Katharina Schramm:** I wanted to touch back on the initial topic of our discussion, which is not just genomics but also justice.

My PhD student Sarah Lempp wrote a great thesis on the hetero-identification committees. She shows the conundrum at their heart–the issue of justice, of racism and how to address it, how to create more equitable relations, how to ensure more access, how to do affirmative action, and so on.

Hetero-identification by phenotype has been scandalised and problematized for good reasons. These panels have been called race tribunals and compared with apartheid and other forums and committees assembled to decide upon someone’s race. But the question of justice is really at the core. Similar to what colleagues here have said about race being used as a proxy for health inequities, these committees use race as a proxy to address injustices, producing new problems, new forms of injustice in turn.

**Ernesto Schwartz-Marin:** We have to discuss more deeply the connection between genomics and justice, especially when it comes to tackling racism. And this goes back to the issue of “strategic essentialism”. The way I understand this concept is that you can, in a sense, only address injustice through the categories that oppress you. How can you tackle racism without, in one way or another, making race real in some respects?

The work of Sarah Lempp is really interesting, because it is a fantastic way to show how race is even made as you look at the pictures people use in the hetero-identification commissions in Brazil (Lempp, 2024). There are so many ways to look at the pictures and provide a phenotypical judgment, which in itself merits attention. I think Pete and Michael Kent have an interesting article in our co-published special issue which addresses these issues in an indirect way (Kent and Wade, 2015). Nevertheless, this question of justice in medicine, justice in forensics and databases needs to be explored more fully.

**Amade M’charek:** I should say, some of you might have heard about the recent elections in the Netherlands and the horrific win of the far-right party of Geert Wilders. He has been sued for racist slogans, motivating audiences to chant that we want fewer Moroccans. This led to a court case in which the judge actually had to determine whether this act was racist. And, of course, it was a racist act, but the judge ruled that it was racism because Moroccans are a “race”—you see what I mean? A national identity here became race. All the judges and legal experts I’ve talked to find this odd, yet argue that we need race in our legislation in order to adjudicate cases of racism and punish them. But in fact, it is a fiction that you need or can precisely define race—so, I find myself arguing against the notion of race in our legislation.

Both the constitutional law and the Criminal Code in the Netherlands use the category of race in ways that are already problematic and contradictory. Constitutional law talks of race as a social construct—how people get racialized and how cases of racism should be prosecuted. Whereas the Criminal Code talks about race as biology in the context of forensic DNA, to determine the race of an unknown suspect for instance. So here we have a slippage between two different kinds of race definitions—race as descriptor of physical biological phenomena and race as social construct.

This is why I’m not at ease with “strategic essentialism”. I do not think we need to be accurate or have clearly defined racial categories (which do not exist!) in order to understand and fight racism, or call out racist behaviour. So, I’m actually more and more shying away from race as a descriptor or even as self-identification. But I’m aware, of course, that the world creates difference all the time, and there are many people who think they are better off embracing a racial identity. This is where it gets complex.

**Ernesto Schwartz-Marin:** And these debates are huge in Latin America. Because we have always had a thorny, horrible relationship with race. In fact, whenever I wanted to have a good workshop or focus group during my field work in Latin America, I dropped the word race. Immediately people would get angry, try to destabilize the word. It was a fantastic approach because they were so resistant.

So when I read Sarah Lempp’s dissertation, I thought this is such a different world from the one I’ve encountered in all my field work in Latin America. The hetero-identification commissions in Brazil—there is nothing even close to that in Mexico. And it is not that we do not have Indigenous ancestry but we have a very thorny relationship with our ancestral roots and even thinking about racial categories is difficult for us.

In terms of strategic essentialism, I think this goes back to a problem with genomics—I think genomics” anti-racist agenda is incredibly colour-blind. Because it says that we are all similar, in very abstract ways. There are lots of issues of justice that such a perspective simply can’t address. So, you have a racialization that lacks teeth, which is a huge problem with genomics in my opinion.

**Andrés Moreno:** An additional thing: Michel raised the issue of empowering local communities to study their own populations and avoid this trend of “scientific colonialism” through genomics. We are seeing a dramatic boosting of genomics in terms of studies, volume, and resources in wealthier nations—those that have the largest biobanks and are generating the most discoveries with the volume of data they have (Bentley et al., 2017; Ruderman, 2023).

So, one aspect of justice is indeed the concepts and population descriptors we’re using, but another is where that research is done, *who* is doing it, and the extent to which you include a diverse array of people, engage with communities, and so on. In fact, I cannot just decide on my own that the better term to describe South African people is X or Y—let’s go and ask them, engage them, and integrate their views. Empowering local communities and ensuring justice is at the heart of this work.

**Ernesto Schwartz-Marin:** I like that point a lot, Andrés, and I appreciate your work in this regard. We would need another session to discuss global North/South dynamics properly, but it is very important.

As you know I’ve studied ideas of “genomic sovereignty” advanced by countries as a decolonial measure, especially in Mexico, and I have a critique there. We tend to think that when we go to the local community, they would give us categories that are more emancipatory. But sometimes that’s not the case. I’ve done a lot of grassroots engagement and racism exists there too, right? When I did field work in Colombia, there were a lot of white supremacists going to genetic ancestry testing. For me, this issue links back to colonialism in practice, and is not just about the categories we use in science. Nevertheless, there is a lot more to discuss here.

**Amade M’charek:** This point of the power imbalance is closely related to our discussion on how race is made and the categories we use. There was this slogan “Knowledge for the West, Genes From the Rest”. Who has a say in deciding which categories matter? Which categories will travel, and which will be blocked? I mean it is larger than that, obviously, but it is there when these categories are taken for granted, the way they are situated within the histories they carry, and how they are reified.

Even scientists in other places in the world who are trying alternative classifications, which are perhaps more representative of local concerns, will not filter through at the international level due this power imbalance in who gets to set the terms, who gets to stabilise the categories. And that is a problem we all might have, whether you are doing your research in Friesland, Netherlands or somewhere in the south of Tunisia with an Indigenous population.

**Ernesto Schwartz-Marin:** That is why I think this is such an interesting topic, and something we need to expand on further. I have to say I enjoyed this roundtable massively. It is difficult to cover all aspects of this topic in one discussion, but we hope this provides a starting point for readers to explore the diverse literature in this field (for example, Duster, 2003; Reardon, 2005; Krimsky and Sloan, 2011; Bliss, 2012; Roberts, 2012; Benjamin, 2013; Nelson, 2017; Anderson and Lindee, 2020; Graves and Goodman, 2021). Thank you so much, everyone. It was a real pleasure engaging with you all.

## Key takeaways

- [A key underlying conundrum] is the concept of “population”, and the way it is defined in social terms but investigated in biological terms” – Peter Wade
- “We should avoid thinking that biogeographic ancestry is a given, it is something that’s “out there”, while “race” is constructed. In the early days of the Human Genome Diversity Project, the late [evolutionary biologist] Allan Wilson argued that all definitions of population are arbitrary [and a lot depends on our approach to sampling] … It is important to pay attention to the way databases have been set up, the way we have clustered people based on existing social, economic, historical, natural borders…. Because this becomes self-reinforcing–we’re first producing a particular kind of “diversity” and then returning to prove it exists” – Amade M’Charek.
- We cannot take categories for granted–they have a historical weight that must be acknowledged. “There are haunting reverberations of classificatory practices, of historical hierarchies, of the violence that surrounds race—not only in terminology, but also the practices around race and race-making. And this is where critical social science has engaged with genomics, tracing out these ghosts.” – Katharina Schramm
- “It is important to note that labels [like European population, African population], even if they are as simple as geographic, are discrete endpoints on a larger gradient, which is what our studies have been discovering. There is no “pure’ population only existing in Africa, Europe, Asia or America” – Andrés Moreno
- [I’ve been part of initiatives] where we’ve been trying to find the best possible word to explain that ancestry is just the output of an algorithm which simplifies a model of grouping people. It is not *really* your “ancestors”, but a description of how your genetic profile is being classified given a certain reference panel” – Andrés Moreno
- “With the pressure to publish and have your genomics papers out in top-notch journals and so forth, the work involved [in thinking about sociopolitical implications] would be seen as something holding you back, taking up precious time and not adding to the “research”. I do not think a book chapter dealing with the issues we’re discussing here around race, which would be time consuming, would be valued in the same way as a technical paper, and therefore funding makes its way into explaining a lot of this pattern.” – Diogo Meyer
- A more just genomics would need geneticists to also think about race as a political category, and consider how they are engaging with its historical weight in their research.
